# The period from prodromal fever onset to rash onset in laboratory-confirmed rubella cases: a cross-sectional study

**DOI:** 10.1186/s12879-021-06158-9

**Published:** 2021-05-15

**Authors:** Tsuyoshi Ogata, Maki Murooka, Makoto Akashi, Akemi Ishitsuka, Akari Miyazaki, Shuuichi Osawa, Kanako Ishikawa, Keiko Tanaka-Taya, Ritei Uehara

**Affiliations:** 1 Itako Public Health Center of Ibaraki Prefectural Government, Itako, 311-2422 Japan; 2 Ryugasaki Public Health Center of Ibaraki Prefectural Government, Ryugasaki, 301-0822 Japan; 3 Tsuchiura Public Health Center of Ibaraki Prefectural Government, Tsuchiura, 300-0812 Japan; 4 Ibaraki Prefectural Institute of Public Health of Ibaraki Prefectural Government, Mito, 310-0852 Japan; 5 National Institute of Infectious Diseases, Tokyo, 162-8640 Japan; 6 Department of Epidemiology for Community Health and Medicine, Kyoto Prefectural University of Medicine, Kyoto, 602-8566 Japan

**Keywords:** Rubella, Fever, Rash, Prodrome, RT-PCR

## Abstract

**Background:**

In resource-limited settings, where rubella is endemic, it is difficult to determine which sporadic case should be tested for rubella. The study aimed to provide useful evidence to help screen rubella cases for real-time reverse transcriptase-polymerase chain reaction (RT-PCR) examination for rubella in resource-limited settings.

**Method:**

Suspected rubella patients identified by a physician and brought to the notice of the Ryugasaki public health center or the Tsuchiura public health center were enrolled from April 2018 through December 2019. The inclusion criterion was a confirmed rubella diagnosis based on laboratory tests. We studied the distribution of the time from the onset of fever until the onset of rash.

**Results:**

The study included 86 cases with simultaneous presentation of fever and rash. Twenty-nine cases had confirmed rubella based on the laboratory diagnosis. Among these, the time from the onset of fever until the onset of rash was limited to − 1 day to 2 days. The number of rubella cases was the highest when the onset of rash was on the following day of the onset of fever. Of the 78 patients who underwent the RT-PCR test, 48% tested positive for rubella among those with a time from the onset of fever to the onset of rash between − 1 day and 2 days (22 out of 46, 95% confidence interval 34–62%); no positive results (0 out of 30, 95% confidence interval - 14%) were seen in patients with a time from fever to rash onset ≥3 days.

**Conclusion:**

The period from the onset of fever to the onset of rash was limited to − 1 day to 2 days among confirmed rubella patients. If the period from onset of fever to the onset of rash was ≥3 days for a patient, the likelihood of rubella was low.

## Background

Rubella is an infectious disease caused by rubella virus. The virus is transmitted through contact with droplets or nasopharyngeal secretions from an infected individual. The incubation period for acquired rubella ranges from 12 to 23 days. The major clinical manifestation is a generalized maculopapular rash beginning on the face and neck and progressing downwards. Transient joint symptoms may occur in adult women. However, symptoms of acquired rubella are generally mild, and a considerable proportion of infected individuals are asymptomatic [1, 2]. After fetal infection, fetal malformation called congenital rubella syndrome (CRS) may develop. Therefore, the prevention of rubella is a significant public health challenge [3].

Universal vaccination with a rubella-containing vaccine (RCV) for susceptible individuals is necessary for eliminating rubella and CRS. In 2018, 168 (87%) of the countries in the world had introduced RCV in their vaccine schedule. However, only 81 WHO member states (42%) have verified eliminating rubella, and 26,006 rubella cases have been reported globally [4]. In Japan, although the goal of eliminating rubella by fiscal year 2020 was set, 2941 and 2306 rubella cases were reported in 2018 and 2019, respectively [5, 6]. Furthermore, four cases of CRS were also reported in 2019 and 2020 [7].

Surveillance with a rapid and exact diagnosis of rubella patients is important for patient and community interventions. However, the clinical diagnosis of rubella is unreliable because clinical manifestations are generally subclinical or nonspecific [8, 9]. Detection of the rubella virus by polymerase chain reaction (PCR), rubella-specific IgM antibody, or a significant rise in the IgG antibody from paired sera provides a reliable diagnosis of acute rubella infection [2, 10, 11]. Although the detection of rubella-specific IgM is the standard method for laboratory diagnosis, rubella may not be detected until 4 days after the onset of the rash [10, 12]. PCR has been extensively evaluated for its usefulness in detecting rubella virus in clinical specimens. Real-time reverse transcriptase (RT)-PCR can be used to detect rubella virus [11, 13]. However, rubella RNA specimens should be collected as soon as possible after the onset of rash, as the sensitivity of RNA detection methods declines considerably 5–7 days after rash onset [14].

While a surveillance system with laboratory confirmation of cases may be important for regional elimination, the investigation of cases becomes very complex and time-consuming in the near-elimination stage [10]. Since many patients develop acute rashes due to other diseases, RT-PCR tests at the early stage of the disease as a public health intervention may impose a heavy burden on the laboratories when rubella is endemic and the resources for testing are limited in the area. It is also difficult to determine the case to prioritize for the rubella test, especially when the case is sporadic without contact or exposure.

In a rubella patient, especially an adult, the prodromal stage precedes the rash by 1 to 5 days. The prodromal symptoms present as fever, malaise, lymphadenopathy, and upper respiratory symptoms. The fever is described as low-grade [2, 15, 16], and < 39 °C [1] in the previous literature. The prodrome symptoms is rare in children. We believe that fever is the most common noticeable symptom in the patient [2, 15, 16]. Data on the period between the onset of fever and the onset of rash in rubella patients may be useful for screening cases that need to be tested for rubella in resource-limited settings. However, to the best of our knowledge, no studies have reported data on this period.

Therefore, in this study, we investigated the period between the onset of fever, a prodromal symptom, and the onset of rash among laboratory-confirmed rubella patients. The study aimed to provide useful evidence to help screen cases for RT-PCR examination for rubella in resource-limited settings.

## Method

### Study design and setting

In this cross-sectional study, patients were recruited from clinics and hospitals in the jurisdiction of the Ryugasaki public health center and Tsuchiura public health center of the Ibaraki Prefectural Government. The jurisdiction of these public health centers is approximately 30 to 70 km from metropolitan Tokyo, and the centers serve a population of approximately 714,000.

### Study subjects

In Japan, based on The Infectious Diseases Control Law (The Law), authorities need to be notified of all rubella cases [5]. The inclusion criterion for the study was if a physician in a hospital or clinic suspected a patient to have an infectious disease with a likely differential diagnosis of rubella and notified the Ryugasaki public health center or the Tsuchiura public health center between April 2018 and December 2019, the patient was enrolled. The relevant public health center implemented an active epidemiological investigation of the patient based on The Law. Public health nurses of the public health centers interviewed the patient and collected data on demographics, symptoms, rubella vaccination history of the patients, and history of definite contact with a rubella patient.

### Rubella confirmation

In this study, contracting rubella was confirmed by laboratory diagnosis. As per the national guidelines for the prevention of rubella, we adopted laboratory diagnosis by RT-PCR test, rubella IgM antibody assay, or IgG antibody measurement from paired sera in the study subjects.

In cases showing the onset of rash no more than 7 days ago or cases recently vaccinated with RCV, a real-time RT-PCR test was performed. Public health nurses collected blood samples, throat swabs, and urine samples and transported them at a temperature of 4 °C to the Institute of Public Health of the Ibaraki prefectural government, where an rubella assay was implemented within 48 h.

Samples were centrifuged. We used commercial kits (Maxwell RSC Viral Total Nucreic Acid Purification Kit: Promega) for the extraction of RNA from the specimens. We performed a one-step assay that combined the reverse transcription and PCR amplification steps in a single reaction using TaqMan Fast Virus 1-step Master Mix and an Applied Biosystem 7500 Fast real-time PCR system designed by Applied Biosystems (AB; Foster City, CA). The primer contained NS (32–54) Fwd and NS (143–160), and the probe contained NS (93–106). The positive and negative controls were run in parallel. If the cycle threshold (Ct) value of the positive control was not more than 40 and the Ct value of the sample was not more than that of the positive control, the result was labeled rubella positive. We also implemented assays of other viruses, including measles and human herpesvirus 6 and 7 (HHV-6 and 7). However, we did not implement a rubella IgM assay and did not collect samples of RT-PCR-negative cases in the convalescent phase for rubella IgM detection.

In this study, most patients with positive rubella IgM antibody or IgG antibody outcomes had already been diagnosed by a clinic or a hospital before the notification and initiation of the epidemiological investigation by the public health center. The assay was implemented at a private laboratory; we were unable to obtain details on the exact method of these tests. We did not implement a PCR test on these cases because more than 7 days had passed since onset of the rash.

### Outcome

The outcome was the period between fever and rash onset. We defined it as the difference between the calendar day of subjective fever onset in the patient (the reference date) and the calendar day of patient-reported rash onset. We excluded subjects who had neither a rash nor fever or did not manifest rash and fever simultaneously.

### Ethical approval

We collected the data as per the regulations governed by The Law. We obtained written informed consent from all the patients or their parents for the study. The study protocol was approved on 25 July 2019 by the Ibaraki Prefecture Epidemiological Research Joint Ethics Review Committee (protocol number: R1–06).

### Statistical analysis

We assessed the outcome of the laboratory tests by various factors, such as sex, age, vaccination history, etc. We also assessed the distribution of time from the onset of fever until the onset of rash by demographic data, history of definite contact with a rubella patient, rubella vaccination history, and laboratory diagnosis results for rubella. Fisher’s exact test was used, and a *p*-value < 0.05 was considered statistically significant. We calculated the percentage of positive cases by the period between the onset of fever and the onset of rash among patients who underwent an RT-PCR test, and among patients who underwent an RT-PCR test and had 0–3 days of interval between rash onset and RT-PCR test. We estimated population mean and 95% confidence interval of the percentage. Statistical analyses were performed using R (version 2.4–0; The R Foundation for Statistical Computing, Vienna, Austria).

## Results

**Fig. 1:**
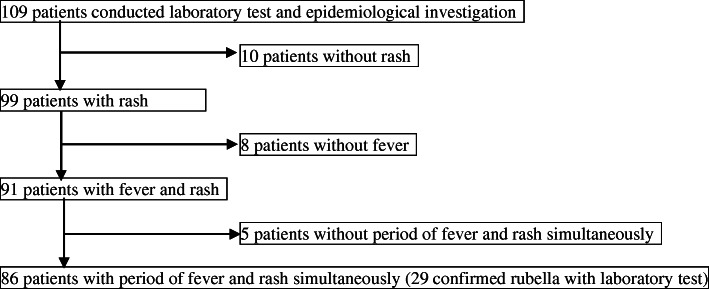
Flowchart of summary

From April 2018 to December 2019, the Tsuchiura public health center and the Ryugasaki public health center conducted an active epidemiological investigation on 109 subjects after being notified by a physician based on The Law. Of these, 10 cases without rash, 8 cases without fever, and 5 cases who had developed a rash after fever alleviation and had no period of simultaneous fever and rash were excluded (Fig. 1). In total, 23 cases were excluded. This study included the remaining 86 subjects with simultaneous fever and rash. Among these, 29 rubella cases were confirmed through laboratory diagnosis (Fig. 1).

Table 1 shows the results of the laboratory test of rubella by sex, age, history of rubella vaccination, history of definite contact with a rubella patient, and methods of laboratory test. Of the 57 cases with a negative RT-PCR outcome for rubella, eight cases were positive for HHV-6 or HHV-7, four for parvovirus, three for EB virus, and two for measles, based on RT-PCR for other viruses.

**Table 1:** Distribution of rubella-positive cases by various factors

Variables	Laboratory test results for rubella			
	N	Positive	Negative	*p*-value
		N (%)	N (%)	
N	86	29 (34%)	57 (66%)	
Sex				
Female	23	1 (4%)	22 (96%)	Reference
Male	63	28 (44%)	35 (56%)	< 0.0003**
Age (years)				
0–29	32	3 (9%)	29 (91%)	Reference
30–59	45	24 (53%)	21 (47%)	0.049 *
60–79	9	2 (22%)	7 (78%)	0.299
Rubella vaccination history				
Never	21	8 (38%)	13 (62%)	Reference
Once	11	4 (36%)	7 (64%)	0.238
Twice	11	0 (0%)	11 (100%)	0.029 *
Unknown	43	17 (40%)	26 (60%)	0.322
History of definite contact of a rubella patient				
Contact	4	4 (100%)	0 (0%)	Reference
Sporadic	82	25 (30%)	57 (70%)	0.011 *
Laboratory test				
RT-PCR	78	22 (28%)	56 (72%)	Reference
Rubella IgM antibody	7	6 (86%)	1 (14%)	< 0.002**
IgG from paired sera	1	1 (100%)	0 (0%)	

*P*-value: Fisher’s exact test; *:< 0.05, **; < 0.01

Table 2 describes the period between the onset of fever until the onset of rash according to the laboratory results for rubella and other factors. In laboratory-confirmed rubella patients, the time from the onset of fever to the onset of rash ranged from − 1 to 2 days with no cases − 2 days or more than 3 days. The number of rubella cases was the the highest when the onset of rash was on the following day of the onset of fever.

**Table 2:** Time between the onset of fever and onset of rash by various factors

	The time from onset of fever until onset of rash (days)									
Factors	-2	−1	0	1	2	3	4	5	6–12	Total
Laboratory test of rubella										
Positive	0	4	10	11	4	0	0	0	0	29
Negative	2	7	10	4	4	15	7	4	4	57
Sex										
Female	0	3	4	3	2	6	2	1	2	23
Male	2	8	16	12	6	9	5	3	2	63
Age (years)										
0–29	2	7	3	0	3	8	5	2	2	32
30–59	0	4	13	12	5	6	2	2	1	45
60–79	0	0	4	3	0	1	0	0	1	9
Rubella vaccination history										
Never	0	4	5	4	3	2	1	0	2	21
Once	0	1	1	3	1	4	0	1	0	11
Twice	1	2	1	0	0	2	3	0	2	11
Unknown	1	4	13	8	4	7	3	3	0	43
History of definite contact of a rubella patient										
Contact	0	2	2	0	0	0	0	0	0	4
Sporadic	2	9	18	15	8	15	7	4	4	82

All values are N

Of the 78 subject cases with RT-PCR test results, 48% (22 out of 46, 95% confidence interval 34–62%) cases with the period from onset of fever to the onset of rash between − 1 day and 2 days were positive, while there were no positive cases (0 out of 30, 95% confidence interval – 14%) in patients with the period from onset of fever to onset of rash ≥3 days (Table 3). The percentage of positive cases was the highest in subjects in whom the onset of fever was followed by the onset of rash the following day (67%).

**Table 3:** RT-PCR test results by the time from onset of fever until onset of rash

			The time from onset of fever until onset of rash (days)								
		−2	−1	0	1	2	3	4	5	6–12	Total
Positive	N (%)	0 (0)	2 (22)	9 (50)	8 (67)	3 (43)	0 (0)	0 (0)	0 (0)	0 (0)	22
	Subtotal^a^	0	22				0				
	(%)	(0)	(48)				(0)				
Negative	N (%)	2 (100)	7 (78)	9 (50)	4 (33)	4 (57)	15 (100)	7 (100)	4 (100)	4 (100)	56
	Subtotal^a^	2	24				30				
	(%)	(100)	(52)				(100)				
Total	N	2	46				30				78

^a^ Subtotal was calculated for cases in whom the time from onset of fever until onset of rash was −2 days, between −1 day and 2 days, and ≥ 3 days

For the 67 cases who underwent the RT-PCR test and with an interval between the onset of rash and RT-PCR test 0–3 days, 45% (18 out of 40, 95% confidence interval 31–60%) cases were positive for rubella test in patients in whom the period between the onset of fever to the onset of rash was between − 1 to 2 days; no positive cases (0 out of 26, 95% confidence interval – 16%) were seen in patients in whom the period from the onset of fever to the onset of rash was ≥3 days (Table 4).

**Table 4:** RT-PCR test results among cases with short interval between rash onset and RT-PCR

		The time from onset of fever until onset of rash (days)									
		-2	-1	0	1	2	3	4	5	6–12	Total
Positive	N (%)	0 (0)	2 (25)	7 (44)	6 (60)	3 (50)	0 (0)	0 (0)	0 (0)	0 (0)	18
	Subtotal^a^	0	18				0				
	(%)	(0)	(45)				(0)				
Negative	N (%)	1 (100)	6 (7)	9 (56)	4 (40)	3 (50)	13 (100)	6 (100)	3 (100)	4 (100)	49
	Subtotal^a^	1	22				26				
	(%)	(100)	(55)				(100)				
Total	N	1	40				26				67

RT-PCR test results by the time from onset of fever until onset of rash in cases with 0–3 days of interval between rash onset and RT-PCR test

^a^ Subtotal was calculated for cases in whom the time from onset of fever until onset of rash was −2 days, between −1 day and 2 days, and ≥ 3 days

## Discussion

In this study on patients with symptoms of both fever and rash simultaneously and laboratory-confirmed diagnosis of rubella, the period from onset of fever to the onset of rash ranged from − 1 to 2 days. The number of rubella cases was the highest when the onset of rash was on the following day of the onset of fever. If the period from onset of fever to the onset of rash was 3 days or more for a patient, the likelihood of rubella was low.

In near-elimination community settings, thorough early case investigations, including high-quality laboratory testing, are important. A rapid and exact diagnosis of patients is essential for appropriate intervention in suspected rubella-infected patients. However, since many patients develop acute rashes due to other diseases, it may be difficult to determine which sporadic case needs to be tested for rubella in settings where rubella is endemic and resources for rubella testing are limited.

Using all available information, such as clinical, epidemiological, and patient data, is essential for case evaluation [10]. In a rubella patient, a prodromal stage precedes the rash by 1 to 5 days. Fever may be the most common prodromal symptom that is noticeable by a patient. The findings of this study indicate that the time from fever onset to rash onset ranges from − 1 to 2 days in cases of rubella, and this is useful for screening cases that need to be tested in resource-limited settings when rubella is endemic.

A study in China reported that the proportion of rubella patients whose prodromal stage lasted 3 days or more was 16.7% [17]. These results indicate that a prodromal period of 3 days or more does not indicate the likelihood of rubella infection and are comparable with the results of the present study. No previous studies have specifically reported on the period between the onset of fever and the onset of rash in rubella cases. Furthermore, there are no studies that indicate that the rash may precede fever in some rubella patients.

Recently, Nomoto et al. reported that conjunctivitis was significantly associated with adult rubella in Japan. Although it might be useful for the early diagnosis of adult rubella, the time of its appearance was not described [18].

In this study, no positive cases were observed in those who received vaccination twice. Thus, this study supports the significance of universal vaccination of RCV. Confirmed rubella cases based on laboratory tests were higher in males than in females, especially in the age group of 30 to 59 years in the study. This may be because only junior high school girls were subjected to rubella vaccination from August 1977 through March 1995 in Japan. The positivity rate of rubella hemagglutination-inhibiting (HI) antibody of sera was generally higher than 90% among females of all ages and males aged 2 years to those in their early 30s in 2017, whereas it was lower than 90% among males in their late 30s to 50s. Therefore, in 2019, the Government of Japan started an immunization project for rubella targeting adult males with low HI antibody titers [5].

Either real-time RT-PCR test, rubella IgM, or IgG antibody assay was used for confirmation of a patient in this study [15]. Early sample collection and adequate transportation are important for PCR tests [10]. While the sensitivity of RNA detection declines considerably after 5–7 days of rash onset, 74% of the suspected rubella cases are RT-PCR positive within 4 days [12, 14]. Assays of rubella-specific IgM antibodies may give false-positive results because of cross-reactive IgM antibodies from other infections, such as enterovirus and adenovirus infections, interference of rheumatoid factor as part of the immune response to parvovirus B19 infection, or in patients with other causes of arthropathy. Therefore, in countries approaching rubella elimination, the positive predictive value of IgM serology decreases [10, 15, 19].

The results in the study are primarily important in low incidence settings, where the number of rubella cases as a proportion of all rash-fever cases is low. Since the number of cases with negative outcomes of rubella tests are affected by epidemics and vaccinations for other infectious diseases in the area, the positive rates of rubella in this study cannot be applied to other populations externally.

This study has several limitations. First, for the calculation of the period, we used calendar days that did not reflect the exact time. Second, serum rubella IgM during the convalescent phase was not tested for RT-PCR-negative cases since it is not easy to collect samples during this phase [12]. Therefore, the cases could not be excluded as rubella negative unless other viruses were confirmed by an RT-PCR test [20]. However, the sensitivity of the RT-PCR test for rubella was considerably high when they were tested within 4 days of rash onset [12]. In this study, most of the RT-PCR-negative cases were tested within 3 days. Therefore, the proportion of false-negative cases might be small; the likelihood of rubella would also be low if the period from onset of fever to the onset of rash was 3 days or more for a patient [12]. Third, since the onset of fever and rash were mainly based on a patient’s subjective symptoms, it may not reflect the exact objective time. Fourth, the number of participants was limited due to the convergence of the rubella epidemic in the region. It would be useful to further investigate more patients in other areas based on the actual time rather than calendar days in future studies.

## Conclusion

The period from the onset of fever to the onset of rash was limited to − 1 to 2 days among laboratory-confirmed rubella patients. The number of rubella cases was the highest when the onset of rash was on the following day of the onset of fever. If the period from onset of fever to the onset of rash was 3 days or more for a patient, the likelihood of rubella was low.

## Data Availability

The datasets used and/or analysed during the current study are available from the corresponding author on reasonable request.

## Abbreviations

CRS: Congenital rubella syndrome
Ct: Cycle threshold
HI: Hemagglutination-inhibiting
The Law: The Infectious Diseases Control Law
PCR: Polymerase chain reaction
RCV: Rubella-containing vaccine
RT: Reverse transcriptase
WHO: World Health Organization
